# Pitx2 in Embryonic and Adult Myogenesis

**DOI:** 10.3389/fcell.2017.00046

**Published:** 2017-05-01

**Authors:** Francisco Hernandez-Torres, Lara Rodríguez-Outeiriño, Diego Franco, Amelia E. Aranega

**Affiliations:** ^1^Cardiac and Skeletal Myogenesis Group, Departmento de Biología Experimental, Universidad de JaénJaén, Spain; ^2^Cardiac and Skeletal Myogenesis Group, Fundación MEDINA Centro de Excelencia en Investigación de Medicamentos Innovadores en AndalucíaGranada, Spain

**Keywords:** *Pitx2*, myogenic precursor cells, embryonic myogenesis, adult myogenesis, satellite cell and regeneration

## Abstract

Skeletal muscle is a heterogeneous tissue that represents between 30 and 38% of the human body mass and has important functions in the organism, such as maintaining posture, locomotor impulse, or pulmonary ventilation. The genesis of skeletal muscle during embryonic development is a process controlled by an elaborate regulatory network combining the interplay of extrinsic and intrinsic regulatory mechanisms that transform myogenic precursor cells into functional muscle fibers through a finely tuned differentiation program. However, the capacity of generating muscle still remains once these fibers have matured. Adult myogenesis resembles many of the embryonic morphogenetic episodes and depends on the activation of satellite cells that have the potential to differentiate into new muscle fibers. *Pitx2* is a member of the *bicoid* family of homeodomain transcription factors that play an important role in morphogenesis. In the last decade, *Pitx2* has emerged as a key element involved in the fine-tuning mechanism that regulates skeletal-muscle development as well as the differentiation and cell fate of satellite cells in adult muscle. Here we present an integrative view of all aspects of embryonic and adult myogenesis in which *Pitx2* is involved, from embryonic development to satellite-cell proliferation, fate specification, and differentiation. Those new *Pitx2* functions on satellite-cell biology might open new perspectives to develop therapeutic strategies for muscular disorders.

## Introduction

Skeletal muscle is a heterogeneous tissue that represents between 30 and 38% of the human body mass (Janssen et al., 2000). It is composed of individual muscle fibers, diversified in size, shape, and contractile protein content, to fulfill the different functional needs of the vertebrate body such as maintaining body posture, locomotor impulse, or pulmonary ventilation. The genesis of skeletal muscle during embryonic development and postnatal life is a process controlled by an extremely elaborate regulatory network that combines the interplay of extrinsic (e.g., morphogens, neurohormonal input, muscle damage, etc.) and intrinsic elements (gene regulatory elements). The intrinsic elements form hierarchical interactions between transcriptional regulators, regulatory RNAs, and chromatin-remodeling factors. In this sense, during embryogenesis, muscle progenitors are specified by the sequential expression of a network of transcription factors composed of PAX3 and PAX7, and the basic helix-loop-helix (bHLH) myogenic regulatory factors (MRFs) MYOD, MYF5, MYF6 (also called MFR4), and MYOG (Bentzinger et al., 2012; Moncaut et al., 2013). In addition, during adult life the skeletal muscle has the ability to resume developmental mechanisms that compensate for the physiological turnover and damage in order to maintain tissue homeostasis (Schmalbruch and Lewis, 2000; Pellettieri and Alvarado, 2007). This adult myogenesis depends on the activation of satellite cells (SCs), that have the potential to proliferate, differentiate, and generate new fibers, or repair existing ones (Chargé and Rudnicki, 2004). It has been well-established that SCs are closely related to progenitors of embryonic origin (Gros et al., 2005; Relaix et al., 2005; Schienda et al., 2006; Hutcheson et al., 2009; Lepper and Fan, 2010). Thus, many similarities have been discovered between prenatal myogenesis and regeneration in the mature skeletal musculature, such as common transcription factors and signaling molecules (Tajbakhsh, 2009).

During the last two decades the homeobox transcription factor *Pitx2* has emerged as a key element in the fine-tuning mechanism that regulates skeletal-muscle development. Concurrently, several recent experimental pieces of evidence point to the role of *Pitx2* in SC biology. Here, we present an integrative view of the role of *Pitx2* in prenatal and adult myogenesis (from embryonic development to SC proliferation), fate specification, and differentiation. Finally we discuss the potential therapeutic use of *Pitx2* in the future.

## Prenatal and adult myogenesis

In vertebrates, skeletal-muscle development is a biphasic process. A primary (embryonic) myogenesis takes place to generate primary muscle fibers, between embryonic day (E) 9.5 and E14.5 in the mouse. This is followed during fetal stages by a secondary myogenesis which gives rise to the bulk of skeletal-muscle fibers present at birth (Kelly and Zacks, 1969; Biressi et al., 2007; Tajbakhsh, 2009; Deries and Thorsteinsdóttir, 2016). All skeletal-muscle cells have the same underlying functions, although their progenitors within the paraxial mesoderm are spread throughout the embryo at the onset of myogenesis. This bears emphasizing since the genetic networks that control myogenesis present differences depending on the location of those myogenic precursors in the embryo.

### Embryonic myogenesis: the trunk and limb muscles

**Figure 1:**
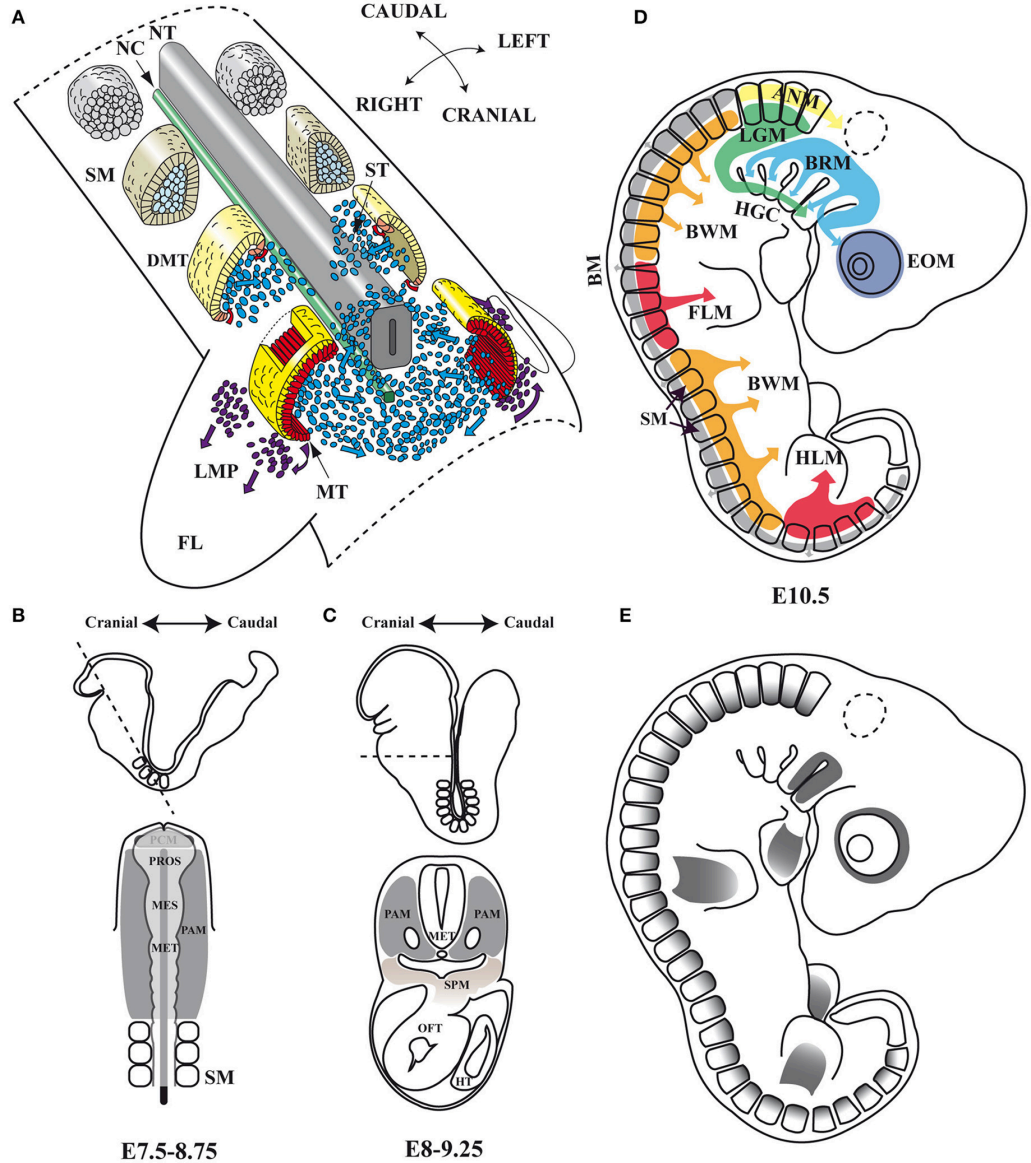
**Embryonic myogenesis (A)** Schematic representation of somite maturation. Somites mature following an anterior to posterior developmental gradient (Modified from *Gray's Anatomy*. The Anatomical Basis of Clinical Practice, 40th Edition Standring, 2008): myogenic precursor cells arise from the epaxial and hypaxial lips of the dermomyotome after archive epithelial-mesenchymal transition (EMT) and migrate toward the limbs to form dorsal and ventral muscle masses where they begin to differentiate. **(B,C)** Head frontal and transverse planes of a mouse embryo between stages of development E7.5–8.75 and E8–9.25 in mouse. At an open neural plate stage, head mesoderm in a frontal plane includes the prechordal mesoderm and the paraxial mesoderm. When the neural tube closes dorsally and the endoderm ventrally, the prechordal mesoderm is integrated within the remaining paraxial mesoderm, which is located anterior to the somites. Dashed line illustrates the cutting plane. **(D)** Origins of skeletal muscles: Myogenic precursors arise from different paraxial mesoderm compartments. **(E)** *Pitx2* expression domains at the E10.5 stage of development in mouse. NT, neural tube; NC, notochord; SM, somites; DMT, dermomyotome; ST, sclerotome; MT, myotome; LMP, limb muscle precursors; FL, forelimb; PAM, head paraxial mesoderm; PCM, prechordal mesoderm; PROS, prosencephalon; MES, mesencephalon; MET, metencephalon; SPM, splanchnic mesoderm; OFT, outflow tract of heart; HT, heart tube; EOM, extra-ocular muscles; BRM, branchial muscles; LGM, laryngoglossal muscles; HGC, hypoglossal cord; ANM, axial neck muscles; BM, back muscles; BWM, body wall muscles; FLM, forelimbs muscles; HLM, hind limbs muscles.

The muscles of the trunk and limbs derive from somites (Figure 1A), which are transient paraxial mesodermal structures that form pairwise on either side of the neural tube, following an anterior-posterior developmental gradient. The somite is initially a spherical unit of polarized epitheloid cells that soon after subdivides into two compartments, the ventral mesenchymal sclerotome and the dorsal epithelial dermomyotome. Shortly afterwards, myogenic precursor cells from the epaxial and hypaxial lips of the dermomyotome undergo an epithelial-mesenchymal transition (EMT) and accumulate underneath, where they differentiate and elongate to form the myocytes of the myotome, the first myogenic structure to develop in the body (Buckingham and Relaix, 2015; Deries and Thorsteinsdóttir, 2016). The epaxial region of the myotome gives rise to the deep back muscles, whereas the hypaxial myotome is the source of body wall muscles and most other trunk muscles (Buckingham and Relaix, 2015; Deries and Thorsteinsdóttir, 2016). In segments adjacent to the limb-region cells of the hypaxial dermomyotome undergo an EMT, leave the epithelial structure, and migrate toward the fore and hind limbs to form dorsal and ventral muscle masses in the limb-bud mesenchyme, where they begin to differentiate and express muscle-specific genes (Biressi et al., 2007; Deries and Thorsteinsdóttir, 2016).

Cell commitment in the somite is highly dependent on a number of transcription factors which act in a hierarchical molecular cascade to orchestrate the specification, determination, and differentiation of myogenic precursors. In the genetic hierarchy that regulates the onset of trunk myogenesis, *Pax3* and *Myf5* play a dominant role (Buckingham and Relaix, 2015). *Pax3* is already transcribed in pre-somitic mesoderm adjacent to the first somite (Schubert et al., 2001) and then throughout the newly formed somites. As somites mature *Pax3* expression becomes confined to the dermomyotome (Goulding et al., 1991) and persists in myogenic progenitor cells that delaminate and migrate from the somite to more distant sites of myogenesis such as the limb (Buckingham and Relaix, 2015). Myogenic cells that have activated the myogenic determination genes *Myf5/Myf6* and *MyoD* downregulate *Pax3* and delaminate from the edges of the dermomyotome (Buckingham and Relaix, 2015). The epaxial myotome then start to form. This depends on the early epaxial activation of *Myf5*, which is driven by *Wnt* and *Shh* signaling, without any *Pax3* and/or *Pax7* requirement (Borello et al., 2006; Buckingham and Relaix, 2015). These cells do not activate *MyoD* but rather *Myog* and differentiate (Kablar et al., 2003). On the other hand, the activation of *Myf5* in the hypaxial somite as well as in the limb depends on PAX3 (Bajard et al., 2006; Buckingham and Relaix, 2015). At this stage MYF6 also acts as a myogenic determination factor (Kassar-Duchossoy et al., 2004). The *Myod* gene is activated after the onset of *Myf5* expression in the rest of the dermomyotome and limbs (Hu et al., 2008). Finally, the transcription factor MYOG is required for the onset of the expression of terminal differentiation genes needed for the fusion of myocytes and the formation of myotubes (Bentzinger et al., 2012).

### Embryonic myogenesis: the head muscles

Although, all skeletal muscle throughout the body originates within paraxial mesoderm, in the head, identifiable compartments such as the somites in the trunk are not evident histologically or by most molecular criteria. This unsegmented head mesoderm is remodeled at the early stages of embryonic development (Figures 1B,C). The unsegmented head mesoderm gives rise to all craniofacial skeletal muscles, which can be cataloged as four distinct populations: extra-ocular (EOMs), branchial, laryngoglossal, and axial neck muscles (Noden and Francis-West, 2006; Tzahor, 2015). EOMs are formed by cells from the cranial paraxial mesoderm that migrate through the first branchial arch (FBA) as well as from the prechordal mesoderm (Jacob et al., 1984; Evans and Noden, 2006; Tzahor, 2015; Figure 1D). Branchial arch muscles are formed mainly by migrating cells from the cranial paraxial mesoderm and the lateral splanchnic mesoderm (Harel et al., 2009; Sambasivan et al., 2009; Tzahor, 2015). Laryngoglossal muscles develop from migratory myoblasts arising from occipital somites that form a condensed mesenchymal band, the hypoglossal cord, which elongates and similarly brings myoblasts ventral to pharynx (Hammond, 1965; Hazelton, 1970; Tzahor, 2015). Finally, in the transition zone between the head and the trunk are the axial neck muscles. They arise from medio-dorsal and latero-ventral domains of occipital and cervical somites (Noden, 1983; Couly et al., 1992; Matsuoka et al., 2005).

The genetic hierarchy governing primary myogenesis in the trunk does not appear to operate for head-muscle formation. Activation of the myogenic program in the head therefore depends on different upstream factors, responds differently to signaling pathways and also displays site-dependent regulation. Branchial-arch-derived muscles depend on *Myf5/Myf6/Myod*, whereas extra-ocular muscle formation is initiated by *Myf5/Myf6* and in their absence cannot be restored by *Myod* (Tajbakhsh and Buckingham, 1999).

### Fetal myogenesis

During fetal myogenesis, secondary fibers in trunk, limbs, and head are generated by the fusion of fetal myoblasts. Secondary fibers form initially at the site of innervation of the primary fiber and are surrounded by the same basal lamina as the primary fiber on which they lie (Duxson et al., 1989). The secondary myotubes remain attached for a short period to primary fibers and subsequently elongate and become independent fibers, which can be distinguished from primary fibers by their relative small size (Kelly and Zacks, 1969). Although, the genetic networks that rule this second stage of prenatal myogenesis is less understood, it is known that the MRFs MYF5, MYOD, and MYOG are also crucial, since in *Myog*^−/−^ as well as *Myf5*^−/−^*:MyoD*^−/−^ double-mutant secondary myogenesis is completely inhibited (Venuti et al., 1995; Kassar-Duchossoy et al., 2004).

### Adult myogenesis

**Figure 2:**
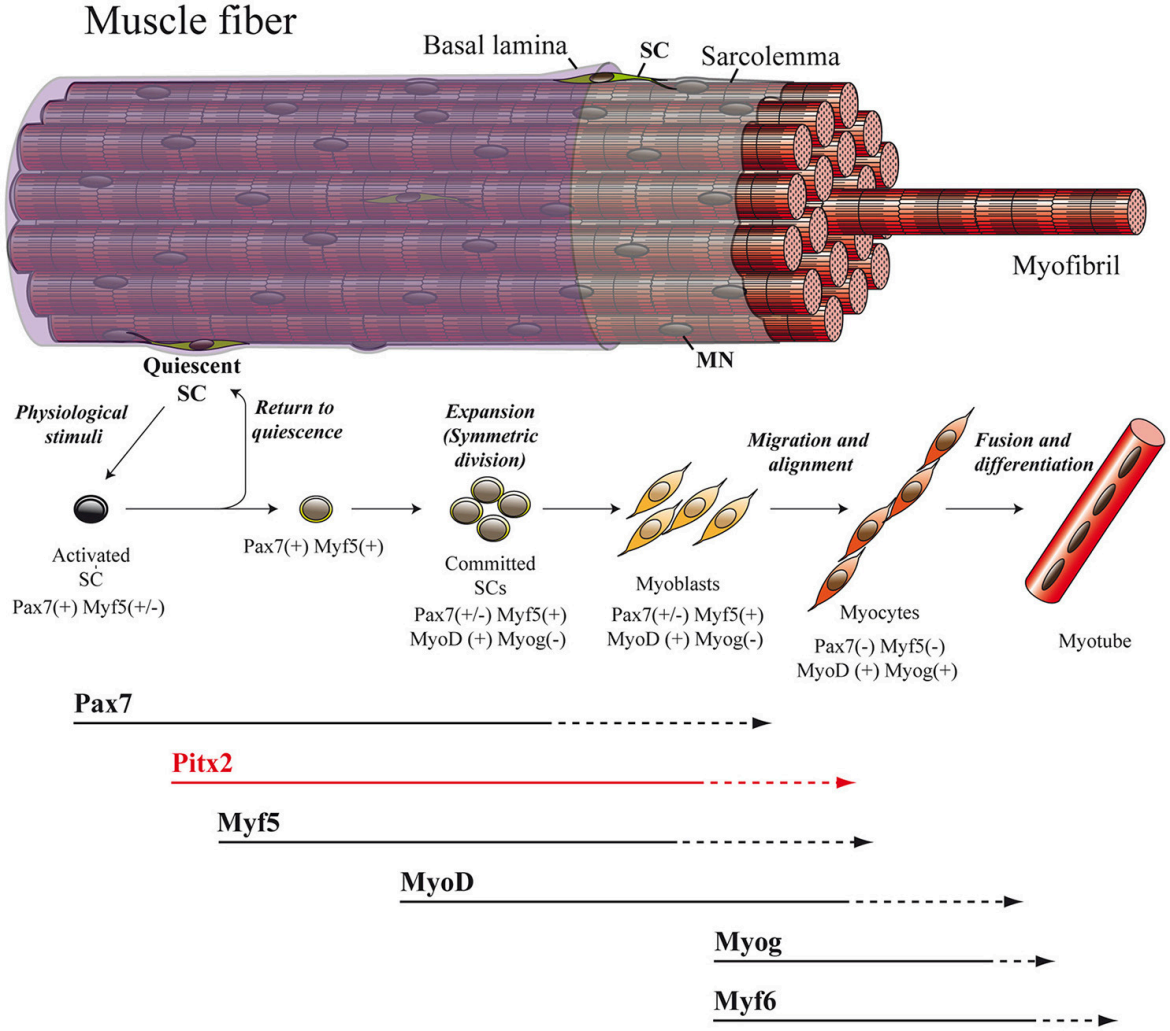
**Adult myogenesis**. The overall myogenic differentiation pathway includes the activation of quiescent SCs, commitment to differentiation, proliferation, fusion to form myotubes and ultimately maturation into myofibers. SC, satellite cell; MN, myonucleus.

The regulatory inputs that orchestrate myogenesis during prenatal myogenesis are partially reactivated in adult muscle repair. In adulthood, the maintenance as well as the repair of muscle tissue are both directed mainly by SCs. These cells, originally identified via electron microscopy in 1961 by Alexander Mauro, are located underneath the basal lamina and adjacent to the plasma membrane of the skeletal-muscle myofiber (Mauro, 1961; Figure 2). In their quiescent state, SC express the transcription factor *Pax7* and represent a genuine stem-cell population indispensable for skeletal-muscle repair (Lepper et al., 2011; Murphy et al., 2011; Sambasivan et al., 2011; Miersch et al., 2017; Stuelsatz et al., 2017). It has been established that SCs in adult muscle represent a lineage continuum of the embryonic myogenic progenitor cells. Thus, while SCs of the body and limbs arise from somites, in common with the muscle that they are associated with (Armand et al., 1983; Gros et al., 2005; Relaix et al., 2005; Schienda et al., 2006), the SCs located in head muscles also originate from the cranial mesoderm (Harel et al., 2009). Within a context of physiological stimuli (physical exercise or pathological conditions) SCs become activated, proliferate, differentiate and fuse to form multinucleated myofibers in order to undergo proper myogenesis (Lepper et al., 2011; Murphy et al., 2011; Sambasivan et al., 2011; Miersch et al., 2017; Stuelsatz et al., 2017; Figure 2). In this regard, numerous studies have revealed a striking similarity between adult and embryonic myogenesis, where the core regulatory network composed of the MRFs MYF5, MYOD, MYOG, and MYF6 is mainly required (Bentzinger et al., 2012; Segalés et al., 2016; Figure 2).

## The *Pitx2* gene

The *Pitx* gene family includes three vertebrate paralogues, *Pitx1, Pitx2*, and *Pitx3*, which have been cloned in multiple organisms (Gage et al., 1999b; Knopp et al., 2013). These three genes encode transcription factors that belong to the *bicoid*-related subclass of homeodomain proteins (Gage et al., 1999b) The members of this family share an almost identical protein sequence within their homeodomains, varying mainly in the N-terminal region (Gage et al., 1999b; Knopp et al., 2013). Mutations or misregulation of *Pitx1, Pitx2*, and *Pitx3* result in developmental disorders in humans, such as Facioscapulohumeral Muscular Dystrophy (FSHD; Dixit et al., 2007), Axenfeld-Rieger syndrome (Semina et al., 1996), and Anterior Segment Mesenchymal Dysgenesis (ASMD; Semina et al., 1998), respectively. Muscle expression of these genes during development has been systematically studied. Thus, *Pitx1* is highly expressed in developing hind-limb-bud mesenchyme and is shown to determine hind-limb identity in mice (Lanctôt et al., 1999; Szeto et al., 1999), chicks (Logan and Tabin, 1999), and fish (Shapiro et al., 2004). On the other hand, *Pitx3* is widely expressed in all skeletal muscles of the head, trunk and limbs (Semina et al., 1998; L'honoré et al., 2007). Curiously, despite its apparent importance in muscle development, the investigation of *Pitx3*^−/−^ mice indicates that *Pitx3* on its own is not required for myogenesis (L'honoré et al., 2007). In this scenario *Pitx2*, the third *Pitx* family member is strongly upregulated and appears to fully compensate for the loss of *Pitx3* during muscle formation (L'honoré et al., 2007). *Pitx2* is also able to control the growth ability of hind-limb mesenchyme together with *Pitx1* (Marcil et al., 2003), indicating the importance of *Pitx2* in the control of skeletal myogenesis during development.

In mice, the *Pitx2* (*Pituitary homeobox 2 or Paired-like homeodomain transcription factor 2*) gene is mapped on chromosome 3 (3G3; 3 57.84 cM) (Gage and Camper, 1997) and is transcribed into three distinct isoforms: *Pitx2a, Pitx2b*, and *Pitx2c*. *Pitx2a* and *Pitx2b* share the same promoter while *Pitx2c* uses an alternative one upstream of exon 4 (Schweickert et al., 2000). In human, *PITX2* is mapped on chromosome 4 (4q25) and maintains a similar genetic structure, but presents a fourth isoform (Arakawa et al., 1998; Cox et al., 2002). This fourth isoform is generated by the *PITX2C* alternative promoter and differential splicing, being able to suppress the transcriptional activity of the other *PITX2* isoforms (Cox et al., 2002). All *Pitx2* isoforms share a K50 DNA-binding homeodomain which binds to the consensus sequence TAATCC (Amendt et al., 1998; Chaney et al., 2005), thus being able to induce a transcriptional activation of *Prl* (Amendt et al., 1998) or *Anf* (Ganga et al., 2003) promoters. The *Pitx2* gene was isolated independently by several research groups and designated as *Otlx2* (Muccielli et al., 1996), *Rieg* (Semina et al., 1996), *Ptx2* (Gage and Camper, 1997), *Brx1* (Kitamura et al., 1997), and *Arp1* (Arakawa et al., 1998). Although, most of these works focused on the role of this gene in the development of brain structures, the authors reported the expression of *Pitx2* in the mesenchyme of the eye, the first and second branchial arches, the fore and hind limbs as well as the dermomyotome at somite stages E8.5 and 10.5 in mouse, and its equivalent stages in chicken (Figure 1E). Soon afterwards, a role for *Pitx2* was also described in left-right asymmetry, being proposed as the molecular transducer of embryonic left-right signaling during early developmental stages at the level of organs such as heart, gut, and/or stomach (Logan et al., 1998; Ryan et al., 1998; Yoshioka et al., 1998; Campione et al., 1999).

## Pitx2 within the genetic hierarchies that control muscle development

### Pitx2 function during embryonic myogenesis

#### Pitx2 in trunk and limb muscle development

The first evidence involving *Pitx2* in the molecular process controlling myogenesis was provided by Kitamura et al. (1999). These authors reported *Pitx2* expression co-localizing in dermomyotomes with *Pax3*, a muscle specification marker playing a key role in delamination and migration of the somitic muscle progenitor cells to the limb buds (Goulding et al., 1994; Tajbakhsh et al., 1997). Later, Marcil et al. demonstrated the presence of PITX2 protein in the myoblasts of the limb bud, displaying an expression pattern similar to that of PAX3 and MYOD (Marcil et al., 2003). All these data suggested that PITX2 was involved in muscle patterning. A more detailed temporal and spatial analysis during initial muscle-cell-cluster formation, by using *lacZ* expression from a *Pitx2* gene insertion, revealed the presence of a *Pitx2*-expressing cell cluster lateral to the dermomyotome (Shih et al., 2007b). This cluster first appeared at the forelimb level at E10.25. After E10.5, Pitx2(+/LacZ)-expressing cells were then also detected on sections of the limbs. Curiously, *Pax3* and the muscle-regulatory factors (MRFs) stained only subsets of *Pitx2*^+^ cells within these areas, and virtually all *Pitx2*^+^ cells in these areas express at least one of these known myogenic markers (Shih et al., 2007b). These observations led the authors to conclude that *Pitx2* marks the muscle lineage more completely than any of the known markers does. In agreement with the interpretation that muscle progenitors express *Pitx2*, L'Honoré et al. (2007) found extensive co-labeling of myotome- and dermomyotome-proliferating cells with PITX2, PAX3, and with PAX7. Notably, they also observed PAX3-positive cells that have completed migration at the proximal limb bud also express PITX2 while not all PITX2-positive cells expressed PAX3. All these data suggest that *Pitx2* might be a player within the molecular pathways controlling muscle-progenitor fate.

**Figure 3:**
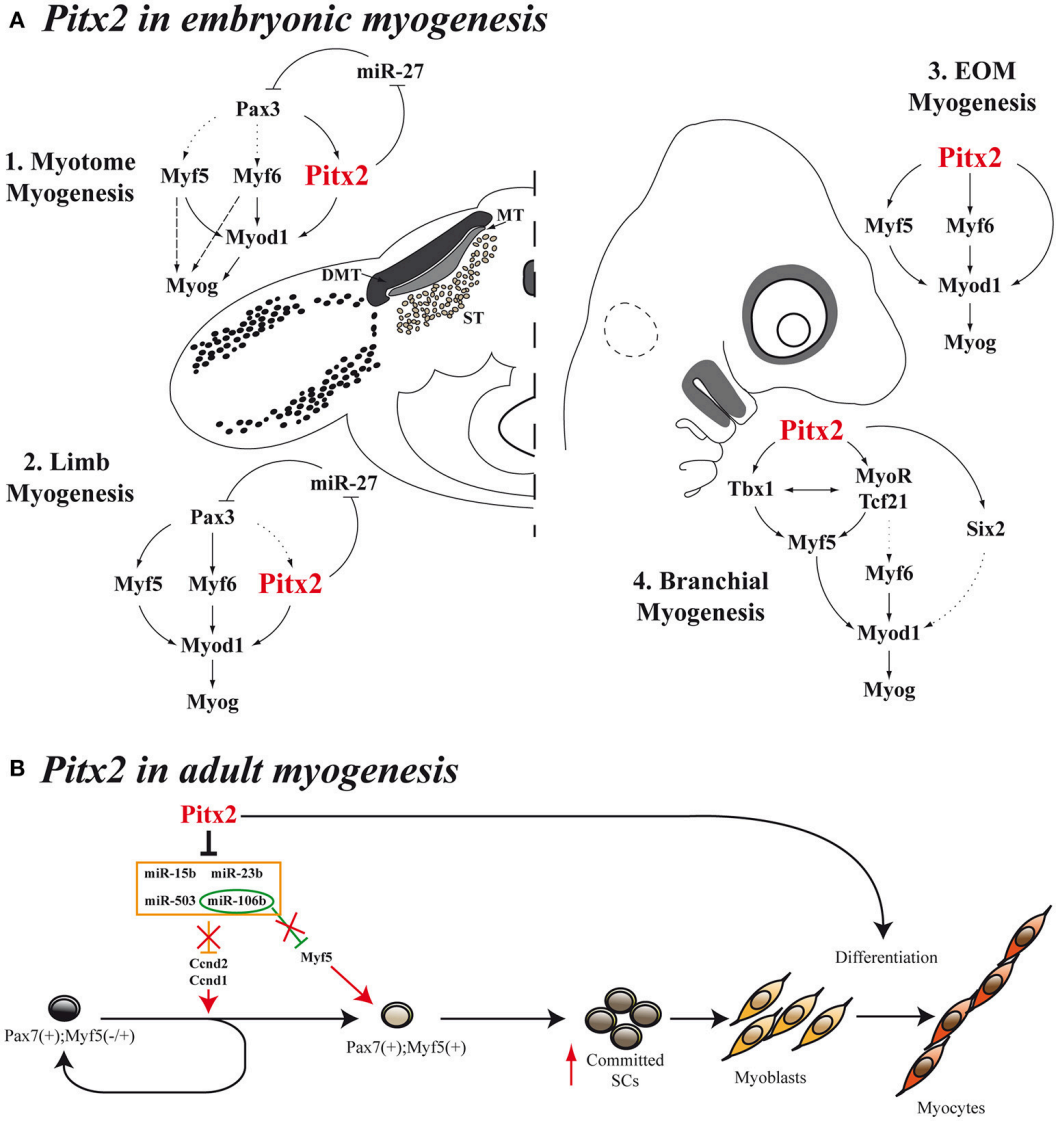
**Models for ***Pitx2*** functions in myogenesis. (A)** During embryonic stages, *Pitx2* contribution is different depending on the initial muscle-cell clusters [myotome myogenesis **(A1)**, limb myogenesis **(A2)**, EOM myogenesis **(A3)**, or branchial myogenesis **(A4)**]. First myocytes of the myotome differentiate through *Myf5* and/or *Myf6* directly to *Myog* without turning on MyoD. This is represented by dashed arrows. Dotted arrows represent direct molecular relationships that still remain elusive **(B)** Proposed model for *Pitx2* in adult myogenesis promoting activation and commitment of SCs.

Sometime afterwards, additional information regarding the hierarchical position occupied by *Pitx2* within the genetic cascade that control somite-derived myogenesis was inferred by using *Pitx2*^−/−^, *Myf5*^*nlacZ*/*nlacZ*^ and *Pitx2*^−/−^*;Myf5*^*nlacZ*/*nlacZ*^ double-mutant mice (L'honoré et al., 2010). In this work, the authors showed that PITX2 protein directly regulates *Myod* expression through binding to its core enhancer in wild-type limbs. In agreement, the authors described a delayed myogenic differentiation and a *Myod* down regulation in *Pitx2*^−/−^ limb buds and proposed that this phenotype appears to be due to the failure to activate the *Myod* core enhancer. However, although the inactivation of *Myf5* and *Myf6 in Myf5*^*nlacZ*/*nlacZ*^ mutant embryos (*Myf6* is inactivated in *cis* in this mutant; Tajbakhsh et al., 1997) did not affect *Myod* expression in limb buds, this inactivation in a *Pitx2*^−/−^ background (*Pitx2*^−/−^*;Myf5*^*nlacZ*/*nlacZ*^) induced a synergic effect that resulted not in a downregulation but in almost a complete loss of *Myod* expression compared with *Pitx2*^−/−^*;Myf5*^*nlacZ*/+^ embryos, where the presence of one active *Myf5* allele prevented *Myod* loss in about 60% of myogenic precursors cells. These results imply that *Myf5* and/or *Myf6* cooperate with *Pitx2* to control *Myod* expression during early limb-bud myogenesis (Figure 3A2). In contrast to limb-muscle cells, myotome expression of *Myod* was not delayed in *Pitx2*^−/−^ embryos. Nevertheless, in *Myf5*^*nlacZ*/*nlacZ*^ mutant embryos, *Myod* expression was delayed by ~2 days. Therefore, the onset of *Myod* expression in the myotome does not appear to depend on PITX2 but mostly on MYF5/MYF6. Nonetheless the inactivation of *Myf5* and/or *Myf6* in a *Pitx2*^−/−^ background (*Pitx2*^−/−^*;Myf5*^*nlacZ*/*nlacZ*^) led to an almost complete loss of *Myod* expression in myotome, as happened in limbs (L'honoré et al., 2010). These results indicate that MYF5 and/or MYF6 also cooperate with PITX2 to control *Myod* expression during myotome development (Figure 3A1).

In addition, it should be stressed that the analysis of *Pax3* mutant *Splotch* mice revealed a deficit of *Pitx2* expression restricted to the myotome (L'honoré et al., 2010). This deficit is not observed in neighboring mesenchyme, indicating that *Pitx2* is downstream of *Pax3* during myotome myogenesis. This is also supported by transcriptome analyses of *Pax3*^*GFP*/+^ *and Pax3*^*GFP*/*PAX*3−*FKHR*^ transgenic mice carried out by Lagha et al. (2010) since, in gain-of-function screens for PAX3 targets, they found an up-regulation of *Pitx2* in somites but not in limb buds. Although, all these seminal works suggest that *Pitx2* could be acting downstream of *Pax3* and in parallel with *Myf5*, at least in the myotome, as noted above, not all PITX2-expressing cells were positive for PAX3, and limb expression of *Pitx2* precedes *Myf5* (L'honoré et al., 2010). Therefore, additional studies using conditional *Pitx2* inactivation in specific myogenic cell populations would help to elucidate the function of Pitx2 in embryonic myogenesis.

Scientific evidence also relates *Pitx2* to cell proliferation in myogenic cells and somite derivates. Notably, *Pitx2* has been reported to be a target gene in the *Wnt/Dvl2*/*beta-catenin* pathway and operates in specific cell types to control proliferation by regulating expression of the growth-control genes *Ccnd1, Ccnd2*, and *c-Myc* (Kioussi et al., 2002; Baek et al., 2003). These authors established that the PITX2 N-terminal domain is required for its effects on proliferation in a myoblast cell line. We have previously demonstrated that *Pitx2c* is the main *Pitx2*-isoform expressed in Sol8 myoblasts and that overexpression of *Pitx2c* in Sol8 cells led to an increase in proliferative capacity and completely blocked terminal differentiation, mainly because high levels of *Pax3* expression were maintained (Martínez-Fernández et al., 2006). Additional data *in vivo* have supported the role of *Pitx2* in cell proliferation during myogenesis. In this sense, Abu-Elmagd et al. (2010) showed that *Pitx2* loss of function in chicken embryos decreased the number of differentiated myocytes/myofibers in the somites, whereas *Pitx2* overexpression increased myocyte/myofiber numbers, particularly in the epaxial region of the myotome. In agreement with Abu-Elmagd et al. and by using *Pitx2c*^−/−^ mutant embryos, we have reported that *Pitx2c* plays a pivotal role in the control of the subtle equilibrium between proliferation and differentiation during trunk and limb myogenesis. This control is exercised by balancing *Pax3*+*/Pax7*+ myogenic population *in vivo* as well as regulating key myogenic transcription factors such as *Pax3* through the repression of *miR-27* (Lozano-Velasco et al., 2011; Figures 3A1,A2). This new function of *Pitx2c* mediated by miRNAs introduces a new level of complexity in the intricate regulatory network that governs myogenesis in the embryo.

#### Pitx2 during head-muscle development

As mentioned above, *Pax3* controls the myogenic specification of muscle embryonic progenitors in trunk and limbs (Tajbakhsh et al., 1997). However, it has been proposed that, instead of *Pax3, Pitx2* plays a major role as an upstream regulator of craniofacial myogenesis (Zacharias et al., 2011; Buckingham and Rigby, 2014). This is supported by the fact that EOM development is impaired in *Pitx2* null mice (Gage et al., 1999a; Kitamura et al., 1999). However, in the early studies it was not evident whether this muscle dysgenesis in *Pitx2* mutant mice resulted from an intrinsic defect in the developing myoblasts or was secondary to the loss of *Pitx2* expression in the periocular mesenchyme. Other authors have subsequently suggested that this phenotype could be due to the *Pitx2* effect on proliferation rate of myogenic precursors (Noden and Francis-West, 2006), in agreement with previously reported data (Kioussi et al., 2002; Martínez-Fernández et al., 2006). The hypothesis that *Pitx2* plays a part in controlling cell proliferation in myogenic cells in this context is also supported by the fact that conditional inactivation of *Pitx2* in neural-crest-derived cells does not affect the early differentiation of eye muscles (Evans and Gage, 2005), while conditional *Pitx2* deletion in the mesoderm induces a down-regulation of *Myf5, Myf6, Myod1*, and *Myog* expression and, therefore, blocks the onset of myogenesis of EOM (Zacharias et al., 2011). In this regard, in 2009, Sambasivan et al. by analyzing double defective mutant mouse embryos *Myf5*(*Myf6*) (*Myf5*^*nlacZ*/+^, *Myf5*^*nlacZ*/*nlacZ*^) and *Myf4*^−/−^ mutants, showed that *Pitx2* cannot ensure survival and activation of *Myod* expression in EOM in the absence of both *Myf5* and *Myf6* (Sambasivan et al., 2009). Shortly afterwards, Zacharias et al. were able to inactivate the expression of *Pitx2* in mesodermal EOM precursors by using a tamoxifen inducible *UBC- CreER*^*T*2^ promoter (Zacharias et al., 2011). This inactivation clearly showed that *Pitx2* is required for EOM precursor specification and survival, acting as an anti-apoptotic factor in the pre-myogenic mesoderm and subsequently activating the myogenic program in these cells through direct binding to *Myf5* and *Myod* promoters (Zacharias et al., 2011). Taken together, all these data clearly suggest that *Pitx2* is an upstream regulator of *Myf5, Myf6*, and *Myod* in EOM embryonic myogenesis (Figure 3A3).

*Pitx2* is also expressed in the myogenic precursors of the FBA. *Tbx1* expression on FBA premyoblast is required for specification leading to *Myf5* and *Myod1* activation in those cells (Kelly et al., 2004). Notably, systemic *Pitx2* mutants, whether *Pitx2*^−/−^ (Dong et al., 2006) or *Pitx2*^*LacZ*/*LacZ*^ (Shih et al., 2007a), display a down regulation of *Tbx1* expression in this structure, although *Pitx2* expression is unaffected in *Tbx1* null mutants (Dong et al., 2006). These data, together with the fact that *Pitx2* directly interacts with *Tbx1* regulatory elements (Shih et al., 2007a) suggest that *Pitx2* is an upstream activator of *Tbx1* in FBA. A fuller analysis of both systemic *Pitx2* mutants reveals that the inactivation of *Pitx2* in FBA results in increased cell death in the mesodermal core and loss of early premyoblast specification markers such as *Six2, Tcf21*, and *MyoR* (Dong et al., 2006; Shih et al., 2007a). Although, the role that *Six2* could play in the myogenesis of the FBA remains elusive, *Tcf21* and *MyoR* are known to be upstream effectors of *Myf5, Myod*, and *Myog* in these initial muscle-cell clusters (Lu et al., 2002). Jointly, these results indicate that *Pitx2* controls the expression of *Myod1* and the onset of myogenesis in FBA through *Tbx1, Tcf21*, and *MyoR* (Figure 3A4).

### Pitx2 during fetal myogenesis

Most of what is known about *Pitx2* concerns early (embryonic) myogenesis. However, a new role for *Pitx2* has recently been unraveled during fetal myogenesis. L'Honoré et al. by using *Pitx2:Pitx3* double conditional mutants, have shown that *Pitx2/3* control the expression of the antioxidant system through the regulation of *Nrf1* and antioxidant enzymes during muscle differentiation (L'honoré et al., 2014a). Thus, *Pitx2/3 depletion* at the onset of differentiation induces an abnormal increase of reactive oxygen species (ROS) levels in differentiating myoblasts and leads to impaired myogenesis due to apoptosis of these cells. These results emphasize the role of *Pitx2* controlling redox conditions during fetal myogenesis.

### Pitx2 is emerging as a key transcription factor that modulates adult myogenesis

During adult life the maintenance and repair of skeletal-muscle tissue is directed by SCs. The regulation of SC function in adults requires the redeployment of many of the regulatory networks fundamental for developmental myogenesis. Although, several efforts have been made during the last few years to disentangle the role of *Pitx2* in embryonic and fetal stages of myogenesis, studies linking *Pitx2* to adult myogenesis have only recently emerged and are still controversial.

The first evidence regarding *Pitx2* expression in SCs was reported by Ono et al. (2010). These authors showed that all *Pitx2* isoforms are expressed in proliferating SC-derived myoblasts. They analyzed SCs with a different ontology, comparing those of the extensor digitorum longus (EDL) of the limb with SCs from the masseter of the head (MAS). They found that *Pitx2b* and *Pitx2c* levels were higher in cells from the EDL than from the MAS, with *Pitx2c* being the main *Pitx2* isoform expressed in proliferating limb SCs (Ono et al., 2010). Based on these distinct gene-expression profiles, the authors suggest that, even after activation and entry into the cell cycle, SCs retain an identity consistent with their ontogeny underlying their distinct properties. Subsequent studies have pointed out that *Pitx2a, Pitx2b*, and *Pitx2c* were expressed at very low levels in proliferating SCs, but increased during the early stages of myogenic differentiation. Meanwhile the constitutive expression of any *Pitx2* isoform suppressed SC proliferation, with the cells undergoing greater myogenic differentiation (Knopp et al., 2013). However, additional evidence underlying the functional relevance of *Pitx2* on SC proliferation has been reported. For example, Herbet et al. demonstrated that *Pitx2* is crucial in maintaining the phenotype of myogenic precursor cells in the extraocular muscles (EOM; Hebert et al., 2013). In this analysis, the authors found that the higher levels of *Pitx2* expression in EOM in comparison with limb muscles were concomitant with longer proliferative state in EOM-derived SCs as compared with limb cells. In addition, the knockdown of *Pitx2* in SCs isolated from EOM slowed their proliferation rate, and a similar trend was seen for SCs isolated from tibialis anterioris muscle. These data led to the conclusion that *Pitx2* helps maintain a proliferating pool of myogenic precursor cells. Finally, the authors highlight that this greater proliferative capacity may facilitate the repair of damaged EOM tissue, thereby contributing to the sparing of EOM in muscular dystrophies (Hebert et al., 2013).

More recently, a study conducted in our laboratory has provided additional information about the molecular mechanisms by which the *Pitx2* transcription factor regulates cell proliferation in SCs (Lozano-Velasco et al., 2015). We have reported that *Pitx2c* expression is higher in early-activated SCs than in long-term activated ones, and our *in vitro Pitx2c* gain-of-function experiments have revealed that *Pitx2c* stimulates *Ccnd1* and *Ccnd2* expression, accelerating cell proliferation during early satellite-cell activation. Moreover, we have demonstrated that such *Pitx2c* effect on SCs proliferation is due to *Pitx2c*-mediated downregulation of the *miRNAs miR-15b, miR-106b, miR-23b*, and *miR-503* (Figure 3B). The existence of the *Pitx2-miRNA* pathway controlling the expression of key regulatory cell-cycle genes in early-activated SCs revealed a role of *Pitx2* in satellite-cell activation. Although, muscle SCs are promising targets for cell therapies, the paucity of SCs that can be isolated or expanded from adult muscle tissue is limiting; thus these findings provide new molecular tools to overcome such a bottleneck. It bears noting that our analyses also showed that *Pitx2c* can increase *Myf5* expression by down-regulating *miR-106b* (Figure 3B), thus expanding the *Myf5*^+^ satellite-cell population and revealing a role for *Pitx2c* in promoting satellite-cell populations more primed for myogenic commitment (Lozano-Velasco et al., 2015). In this context it should be highlighted that in several muscular disorders such as muscular dystrophies, the progressive muscle wasting and weakness is often associated with exhaustion of muscle-regeneration potential. Therefore, the progressive loss of muscle mass has been attributed, at least partly, to the inability of muscle stem cells to efficiently regenerate tissue loss as the result of the disease (Berardi et al., 2014). Thus, critical for the development of effective strategies to treat muscle disorders is the optimization of approaches targeting muscle stem cells and capable of regenerating tissue loss as the result of the disease or as the result of normal muscle turnover (Bertoni, 2014). Notably, very recent reports have been pointed out that muscle stem cells should be considered as a therapeutic target for restoring muscle function in individuals with DMD (Chal et al., 2015; Dumont et al., 2015). Therefore, identification of new *Pitx2* functions in the context of SC biology may significantly contribute to the clarification of the molecular and cellular mechanisms of skeletal-muscle regeneration and may help to develop therapeutic strategies for muscular disorders.

Notably, the analysis of adult single and double *Pitx2:Pitx3* conditional mutant mouse lines targeted to the muscle stem-cell compartment revealed that double mutant SCs undergo senescence with impaired regeneration after injury, suggesting that Pitx2-mediated changes in ROS levels are required for differentiation of SCs (L'honoré et al., 2014b).

All these data provide new insight into the function of *Pitx2* in the molecular mechanisms that control SC behavior and might thus have future application to enhance the regenerative capacity of these myogenic precursor cells. Further analysis using *in vivo* models could aid in understanding how the *Pitx2*-mediated effects on SCs can influence the kinetics of muscle regeneration.

## Conclusions and future challenges

The data reviewed above show that *Pitx2* is a comprehensive marker for cells undergoing myogenic progression, more so than any of the MRFs. This supports models that include a *Pitx2*-dependent pathway in virtually all skeletal muscles. Many pieces of experimental evidence have pointed out that *Pitx2* is the first molecular signal specifying all myogenic precursors in the head muscles. However, although several works have characterized *Pitx2* as a key transcription factor in the molecular cascade regulating trunk- and limb-muscle progenitors, additional work is needed to elucidate the function of *Pitx2* in specification vs. determination during trunk and limb myogenesis. In addition, since seminal works have revealed that *Pitx2* functions on myogenic cells may be due to *Pitx2*-mediated regulation of miRNAs, the role of *Pitx2* in the post-transcriptional control of myogenesis should be further explored.

In parallel, the role of *Pitx2* during adult myogenesis is beginning to be explored. Skeletal muscle has the ability to repair and regenerate due to the presence of resident SCs. SC function in adults requires redeployment of many of the regulatory networks fundamental to developmental myogenesis. Currently, SCs are considered potential therapeutic targets for restoring muscle function in muscle degenerative disorders such as muscular dystrophies. Recent works indicate that *Pitx2* is expressed in proliferating SCs and can promote differentiation of satellite-cell-derived myoblasts. Moreover, the identification of *Pitx2-miRNA* pathways that regulate satellite-cell behavior as well as the impact of Pitx2 on redox condition during satellite-cell differentiation may open insights toward future applications to modulate satellite-cell fate during muscle regeneration. Therefore, these findings propose *Pitx2* as a new player on skeletal-muscle satellite-cell biology and may help to develop therapeutic strategies for muscular disorder.

## Author contributions

FH, LR, and AA conceived of the structure and content. FH wrote the first draft document. FH and LR designed and produced the figures. LR and DF critically revised the manuscript for intellectual content. AA corrected, edited, and approved the final version of the document to be published.

### Conflict of interest statement

The authors declare that the research was conducted in the absence of any commercial or financial relationships that could be construed as a potential conflict of interest.
